# Widespread colonisation of Tanzanian catchments by introduced *Oreochromis* tilapia fishes: the legacy from decades of deliberate introduction

**DOI:** 10.1007/s10750-018-3597-9

**Published:** 2018-04-04

**Authors:** Asilatu Shechonge, Benjamin P. Ngatunga, Stephanie J. Bradbeer, Julia J. Day, Jennifer J. Freer, Antonia G. P. Ford, Jonathan Kihedu, Tabitha Richmond, Semvua Mzighani, Alan M. Smith, Emmanuel A. Sweke, Rashid Tamatamah, Alexandra M. Tyers, George F. Turner, Martin J. Genner

**Affiliations:** 1 Department of Aquatic Sciences and Fisheries, University of Dar es Salaam, P.O. Box 35064, Dar es Salaam, Tanzania; 2 Tanzania Fisheries Research Institute (TAFIRI), P.O. Box 9750, Dar es Salaam, Tanzania; 3 School of Biological Sciences, University of Bristol, Life Sciences Building, 24 Tyndall Avenue, Bristol, BS8 1TQ UK; 4 Department of Genetics, Evolution and Environment, University College London, Darwin Building, Gower Street, London, WC1E 6BT UK; 5 School of Biological Sciences, Bangor University, Bangor, Gwynedd LL57 2UW UK; 6 Department of Life Sciences, Centre for Research in Ecology, Whitelands College, University of Roehampton, Holybourne Avenue, London, SW15 4JD UK; 7 Evolutionary and Environmental Genomics Group, School of Environmental Sciences, University of Hull, Hull, HU5 7RX UK

**Keywords:** Cichlid, Invasive species, Aquaculture, Capture fisheries, Tilapia, Oreochromis

## Abstract

From the 1950s onwards, programmes to promote aquaculture and improve capture fisheries in East Africa have relied heavily on the promise held by introduced species. In Tanzania these introductions have been poorly documented. Here we report the findings of surveys of inland water bodies across Tanzania between 2011 and 2017 that clarify distributions of tilapiine cichlids of the genus *Oreochromis*. We identified *Oreochromis* from 123 sampling locations, including 14 taxa restricted to their native range and three species that have established populations beyond their native range. Of these three species, the only exotic species found was blue-spotted tilapia (*Oreochromis leucostictus*), while Nile tilapia (*Oreochromis niloticus*) and Singida tilapia (*Oreochromis esculentus*), which are both naturally found within the country of Tanzania, have been translocated beyond their native range. Using our records, we developed models of suitable habitat for the introduced species based on recent (1960–1990) and projected (2050, 2070) East African climate. These models indicated that presence of suitable habitat for these introduced species will persist and potentially expand across the region. The clarification of distributions provided here can help inform the monitoring and management of biodiversity, and inform policy related to the future role of introduced species in fisheries and aquaculture.

**Electronic supplementary material:**

The online version of this article (10.1007/s10750-018-3597-9) contains supplementary material, which is available to authorized users.

## Introduction

In Africa, inland aquaculture is a rapidly growing food sector (FAO, 2016), but one of the major consequences of expansion of aquaculture can be the associated spread of cultured species into non-native ecosystems (Naylor et al., 2001), which has led to detrimental effects for many local habitats (Ehrenfeld, 2010; Gichua et al., 2014). Among the most widely cultured groups of freshwater fish species are tilapiine cichlids. They have been introduced to over 140 countries, and established feral populations in at least 114 of these (Deines et al., 2016). The spread to natural habitats from culture facilities has been both unintentional, with individuals escaping from aquaculture facilities (Canonico et al., 2005), and deliberate, with tilapia being released into natural water bodies to improve capture fisheries (Canonico et al., 2005; Genner et al., 2013). Spread of tilapia species into non-native habitats has resulted in negative ecological effects on native species and their habitats through competition and habitat alteration (Canonico et al., 2005). It has also resulted in the loss of unique population genetic structure through hybridisation (D’Amato et al., 2007). Where studies have been undertaken, the ecological impacts on native species are generally perceived to be negative, but ecosystem services provided have been perceived to be positive where they make large contributions to economic income (Deines et al., 2016). Thus, when tilapia introductions are being considered, benefits need to be evaluated in light of potential ecological and economic costs.

**Fig. 1:**
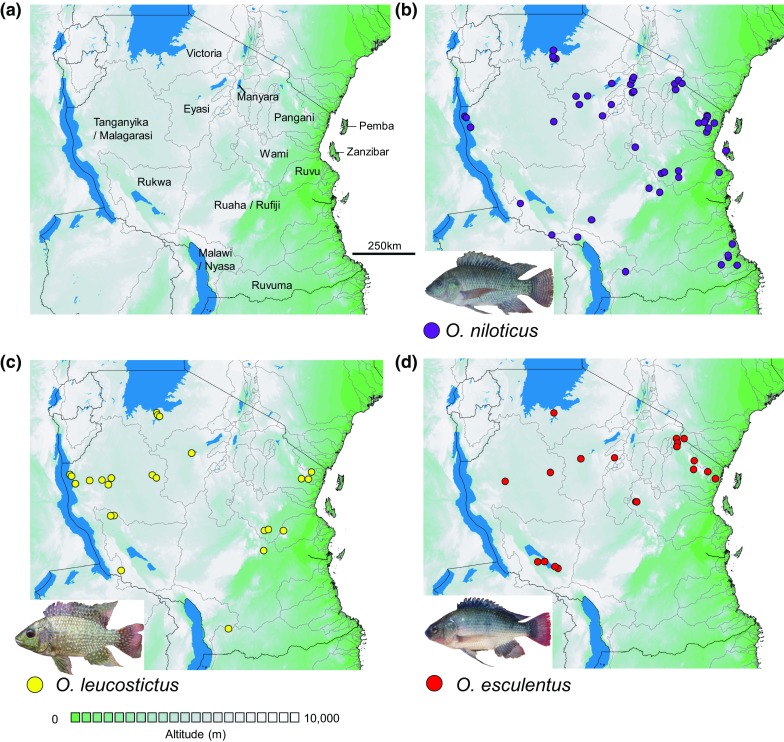
**a** Major watersheds of Tanzania, and **b**–**d** the distribution of species introduced beyond their native ranges (*O. niloticus*, *O. esculentus* and *O. leucostictus*). See Supplementary Information 1 for sampling locations and coordinates

Tanzania has a rich freshwater fish fauna over 630 described fish species (Darwall et al., 2005) spanning eight major freshwater ecoregions (Abell et al., 2008). Although much of this species richness is restricted to the Great Lakes of Malawi, Tanganyika and Victoria (Darwall et al., 2005), over 300 described species have been recorded from other water bodies (Eccles, 1992). Tilapiine cichlids of the genus *Oreochromis* are typically abundant in lakes and slow flowing rivers across the country. In the most recent field guide (Eccles, 1992), 23 *Oreochromis* species were listed, and 21 of these still considered valid *Oreochromis* species [Eschmeyer (2017); Fig. 1)]. Several of these species are significant species of inland capture fisheries (Bwathondi & Mwamsojo, 1993), particularly the introduced Nile tilapia *Oreochromis niloticus* (L.) in Lake Victoria. However, although the introduction has been successful in terms of fisheries production, it may have precipitated loss of native tilapiine cichlid species from much of their former range (Ogutu-Ohwayo, 1990; Witte et al., 1991).

Since the 1990s, landings from capture fisheries in Tanzania have remained stable at approximately 350,000 tonnes (FAO, 2017). Aquaculture is now seen as the potential solution to meeting the increased demand for fish that will accompany a growing human population (Tanzania Government, 2010). Nile tilapia is a favoured species for aquaculture expansion in Africa due to its growth performance, suitability for aquaculture, marketability and stable market prices. The species has also been subject to genetic improvement techniques which could improve yield (e.g. Ponzoni et al., 2011). However, the species can be invasive, and has had detrimental effects on native species at multiple locations in Africa (D’Amato et al., 2007; Zengeya et al., 2013), and elsewhere in its introduced range (Canonico et al., 2005). Thus, from the perspective of balancing conservation with expanding aquaculture, one possibility is that future initiatives could be based on large-bodied native species, with aquaculture species zoned according to which species are native to specific catchments (Lind et al., 2012). Such large-bodied species could include, for example, *Oreochromis urolepis* (Norman 1922), *Oreochromis shiranus* Boulenger 1897 and *Oreochromis jipe* (Lowe 1955) (Table 1). One limitation of this approach, however, has been the limited information available on the current distributions of both the native species or introduced species in Tanzania (Lind et al., 2012).

**Table 1:** *Oreochromis* species in Tanzania considered in this study, focussing on those sampled between 2011 and 2017

Species^a^	Common name	Maximum standard length (cm)^b^	IUCN status	Native range	Exotic/translocated in Tanzania
Species samples					
*O. esculentus* (Graham 1928)	Singida tilapia	50.0	Critically endangered	Lake Victoria basin	Translocated
*O. leucostictus* (Trewavas 1933)	Blue-spotted tilapia	23.2^c^	Least concern	Lakes Edward, George, Albert	Exotic
*O. niloticus* (Linnaeus 1758)	Nile tilapia	60.0	Not assessed	Nile, West Africa, Lake Tanganyika	Translocated
*O. placidus* (Trewavas 1941)	Black tilapia	35.5	Least concern	Ruvuma basin	–
*O. rukwaensis* (Hilgendorf & Pappenheim 1903)	Rukwa tilapia	33.0	Vulnerable	Rukwa and upper Great Ruaha	–
*O. shiranus* Boulenger 1897	Shire tilapia	39.0	Not assessed	Lake Malawi basin	–
*O. urolepis* (Norman 1922)	Wami tilapia	44.0	Not assessed	Coastal Tanzania rivers and islands	–
*O. jipe* (Lowe 1955)	Jipe tilapia	50.0	Critically endangered	Pangani basin	–
*O. amphimelas* (Hilgendorf, 1905)	Manyara tilapia	28.0	Endangered	Central Tanzania lakes	–
*O. korogwe* (Lowe 1955)	Korogwe tilapia	20.8	Least concern	Zigi and Pangani basins	–
*O. variabilis* (Boulenger 1906)	Victoria tilapia	30.0	Critically endangered	Lake Victoria basin	–
*O. chungruruensis* (Ahl 1924)	Chungruru tilapia	19.0	Critically endangered	Lake Kyungululu	–
*O. karomo* (Poll 1948)	Karomo	28.0	Critically endangered	Malagarasi watershed	–
*O. tanganicae* (Günther 1894)	Tanganyika tilapia	42.0	Least concern	Lake Tanganyika basin	–
*O. malagarasi* Trewavas 1983	Malagarasi tilapia	19.7^c^	Least concern^d^	Malagarasi watershed	–
*O. hunteri* Günther 1889	Lake Chala tilapia	25.3^c^	Critically endangered	Lake Chala	–
*O.* “crater lake chambo”	–		Not assessed	Lake Malawi basin	–
Species not sampled					
*O. spilurus* (Günther 1894)	Sabaki tilapia	19.2	Not assessed	East flowing rivers Kenya/Somalia	Potentially exotic
*O. lidole* (Trewavas 1941)	Chambo	38.0	Endangered	Lake Malawi basin	–
*O. karongae* (Trewavas 1941)	Chambo	38.0	Endangered	Lake Malawi basin	–
*O. squamipinnis* (Günther 1864)	Chambo	36.0	Endangered	Lake Malawi basin	–

^a^Listed in Eccles. NB *Oreochromis saka* (Lowe 1953) was listed in Eccles (1992); however, following Turner (1996) we consider this be a synonym of *O. karongae*

^b^Data from Fishbase (Froese & Pauly 2017), unless indicated

^c^Trewavas (1983)

^d^Assessed as *Oreochromis upembae* (Thys van den Audenaerde 1964)

Here we contribute information on the present distributions of *Oreochromis* species across Tanzania, based on fieldwork conducted between 2011 and 2017 across all major catchments in the country. We report these as either native (naturally found in catchment), translocated (species is naturally from Tanzania, but introduced into the catchment) or exotic (naturally found only outside Tanzania, but introduced into Tanzania and the catchment), following the definitions in Copp et al. (2005). We also highlight a case where translocations of Nile tilapia have taken place to part of the Malagarasi catchment that was not known to be naturally occupied by the species. We combine these data with projections to predict suitable habitat for the translocated and exotic species, in current conditions and those projected under future climate regimes. These data build on earlier work on tilapia distributions (Trewavas, 1983; Eccles, 1992), and help clarify the current distributions. Collectively our results demonstrate an unexpectedly wide distribution of introduced species in Tanzania, and highlight the scope for their further range expansion.

## Methods

### Biodiversity surveys

Sampling between July 2011 and September 2017 covered inland water bodies in all major catchments of Tanzania, including the following larger systems: Lake Eyasi, Lake Manyara, Lake Victoria, Lake Malawi/Nyasa, Lake Tanganyika/Malagarasi, Pangani, Rovuma, Ruvu, Rufiji, Wami. We also surveyed four sites on the island of Zanzibar (Fig. 1). Samples of tilapia were collected using one or more of four methods. (1) Deployment of monofilament multimesh gill nets. Each net was 30 m long with a stretched height of 1.5 m, this comprised 12 panels each 2.5 m long and with a stretched height of 1.5 m. Mesh sizes for panels were in the following order 43, 19.5, 6.25, 10, 55, 8, 12.5, 24, 15.5, 5, 35 and 29 mm. (2) Deployment of monofilament single panel gillnets. Each net was 30 m in length, 1.5 m high and had either 50 mm or 60 mm mesh. (3) Deployment of a beach seine, measuring 30 m in length, 1.5 m in height with 25.4 mm mesh and fine mesh cod end. (4) Opportunistic purchasing from artisanal fishers or markets, if the source of fish is known. Fishing methods and effort expended differed among locations depending on water depth, specific habitats characteristics, including the accessibility of the sites at the time of sampling. Our primary aim was to map the distributions using only information on species presence. Thus, we did not exhaustively conduct repeat sampling at the same locations to identify rarer occurrences, and the resulting data are not interpreted here as evidence of species absence.

At each location, sampled individual tilapiines were identified in the field and photographed. Identifications were based on pre-existing field guides and taxonomic treatments (Trewavas, 1983; Eccles, 1992; Seegers, 1996; Turner, 1996). Where possible, individual whole fish were pinned, labelled and preserved. Fish were processed in the field using one of the two methods: (i) field-fixed in dilute formalin (10%), and later transferred to 70% ethanol for long-term storage; (ii) field-fixed in 99% ethanol, and later transferred to 70% ethanol for long-term storage. Geographical coordinates were taken in situ at collection sites using a handheld GPS. Species distribution data were mapped using DIVA-GIS 7.5 (http://www.diva-gis.org), against a background digital elevation map for Africa with 30 s resolution from HydroSHEDS (Lehner et al., 2008). Catchment boundaries were mapped using a Basin outlines shapefile with 15 s resolution, also from HydroSHEDS. This boundary information was used to inform catchments referred to in this study (Table 2). Waterbodies were mapped with the Africa Water Bodies shapefile from the RCMRD Geoportal (http://servirportal.rcmrd.org/), and countries were mapped with the Africa Countries shapefile from ArcGIS (https://www.arcgis.com/).

**Table 2:** The number of locations surveyed in catchments across Tanzania, and the number of locations where each species was recorded

Catchment/species	Survey locations	*O. esculentus*	*O. leucostictus*	*O. niloticus*	*O. placidus*	*O. rukwaensis*	*O. shiranus*	*O. urolepis*	*O. jipe*
Major catchments									
Lake Eyasi	4	1	1	4	0	0	0	0	0
Lake Malawi	12	0	1	2	0	0	7	0	0
Lake Manyara	3	0	0	2	0	0	0	0	0
Lake Rukwa	13	4	3	1	0	10	0	0	0
Lake Victoria	5	1	3	4	0	0	0	0	0
Pangani River	14	7	3	11	0	0	0	0	7
Pemba Island	4	0	0	0	0	0	0	4	0
Ruaha/Rufiji River	14	0	1	3	0	4	0	8	0
Ruvu River	3	0	1	1	0	0	0	3	0
Ruvuma River	6	0	0	0	6	0	0	0	0
Tanganyika/Malagarasi	12	2	10	4	0	0	0	0	0
Wami River	9	2	2	3	0	0	0	7	0
Zanzibar Island	4	0	0	1	0	0	0	3	0
Minor catchments									
Dar-es-Salaam	1	0	0	1	0	0	0	0	0
Lake Kitele	1	0	0	1	0	0	0	0	0
Lake Basotu	1	1	0	0	0	0	0	0	0
Lake Burungi	2	0	0	2	0	0	0	0	0
Lake Chala	1	0	0	0	0	0	0	0	0
Lake Mansi	1	0	0	0	0	0	0	1	0
Lake Singida	1	0	0	1	0	0	0	0	0
Lake Sulungali	1	0	0	1	0	0	0	0	0
Lukuledi River	3	0	0	1	2	0	0	0	0
Mbwenkuru River	2	0	0	1	0	0	0	2	0
Miteja River	1	0	0	0	0	0	0	1	0
Mlingano Dam	1	0	0	0	0	0	0	0	0
Rutamba lakes	3	0	0	3	0	0	0	0	0
Zigi River	1	1	0	1	0	0	0	0	0
Total	123	19	25	48	8	14	7	29	7

Catchment/species	*O. amphimelas*	*O. korogwe*	*O. variabilis*	*O. chungruruensis*	*O. karomo*	*O. tanganicae*	*O. malagarasi*	*O. hunteri*	*O. “*crater lake chambo”
Major catchments									
Lake Eyasi	2	0	0	0	0	0	0	0	0
Lake Malawi	0	0	0	1	0	0	0	0	6
Lake Manyara	1	0	0	0	0	0	0	0	0
Lake Rukwa	0	0	0	0	0	0	0	0	0
Lake Victoria	0	0	1	0	0	0	0	0	0
Pangani River	0	1	0	0	0	0	0	0	0
Pemba Island	0	0	0	0	0	0	0	0	0
Ruaha/Rufiji River	0	0	0	0	0	0	0	0	0
Ruvu River	0	0	0	0	0	0	0	0	0
Ruvuma River	0	0	0	0	0	0	0	0	0
Tanganyika/Malagarasi	0	0	0	0	3	3	8	0	0
Wami River	0	0	0	0	0	0	0	0	0
Zanzibar Island	0	0	0	0	0	0	0	0	0
Minor catchments									
Dar-es-Salaam	0	0	0	0	0	0	0	0	0
Lake Kitele	0	0	0	0	0	0	0	0	0
Lake Basotu	0	0	0	0	0	0	0	0	0
Lake Burungi	0	0	0	0	0	0	0	0	0
Lake Chala	0	0	0	0	0	0	0	1	0
Lake Mansi	0	0	0	0	0	0	0	0	0
Lake Singida	1	0	0	0	0	0	0	0	0
Lake Sulungali	1	0	0	0	0	0	0	0	0
Lukuledi River	0	0	0	0	0	0	0	0	0
Mbwenkuru River	0	0	0	0	0	0	0	0	0
Miteja River	0	0	0	0	0	0	0	0	0
Mlingano Dam	0	1	0	0	0	0	0	0	0
Rutamba lakes	0	3	0	0	0	0	0	0	0
Zigi River	0	1	0	0	0	0	0	0	0
Total	5	6	1	1	3	3	8	1	6

### Modelling habitat suitability for introduced species

Records obtained during our sampling efforts between 2011 and 2017 found three species had been introduced beyond their native range *O. niloticus, Oreochromis esculentus* (Graham 1928) and *Oreochromis leucostictus* (Trewavas 1933). We modelled suitable habitat for these species to determine if their limited spread had been linked to environmental variables, and to identify areas that could potentially be colonised with further introductions. Bioclimatic environmental data were obtained at a downscaled 2.5 arc minute spatial resolution using Worldclim v.1.4 (Hijmans et al., 2005), and the variables used were limited to temperature and precipitation for “current conditions”, representative of the time period 1960–1990. The variables included annual trends (mean annual temperature, annual precipitation) and limiting environmental factors (temperature of the coldest and warmest months, and precipitation of the wettest and driest months), namely Bio1 = annual mean temperature, Bio5 = maximum temperature of the warmest month, Bio6 = minimum temperature of the coldest month, Bio12 = annual precipitation, Bio13 = precipitation of wettest month and Bio14 = precipitation of driest month. We also included elevation, as this can represent a proxy for numerous environmental variables (Koerner, 2007). We note that they will not be able to identify key local limiting factors in determining distributions, for example, water flow rates, substrate, shelter and the abundance of prey, predators and parasites. However, the use of bioclimate variables across such large spatial scales is justified as (i) bioclimate air temperature variables correlate closely with in situ measurements of water temperature (Domisch et al., 2015), and (ii) bioclimate variables can act as reliable predictors of abundance of freshwater species (Knouft & Anthony, 2016).

Future climate data for the years 2050 (2041–2060) and 2070 (2061–2080) were obtained from some of the most recent climate projections used by the IPCC Fifth Assessment Report (IPCC, 2013). Specifically, we used two Global Climate Models (ACCES-1.0, CSIRO-BOM, Australia; MIROC-ESM, Centre for Climate Research, Japan) simulated under two Representative Concentration Pathways (RCPs; RCP 4.5, RCP 8.5). These two RCPs were chosen as they represent very different emission scenarios whereby CO_2_ emissions have stabilised without overshoot to ~ 650 ppm by 2100 (RCP 4.5) or have continued to rise under the current trajectory to ~ 1,370 ppm by 2100 (RCP 8.5) (Moss et al., 2010). We used Worldclim v.1.4 to source the relevant Bioclim variables for the two climate models and emission scenarios. Data were downloaded at 2.5 arc minute spatial resolution, and cropped using the R package Raster (Hijmans, 2015) to longitude 25°E to 42°W, and latitude − 18°S to 5°N.

Ecological niche models of environmental suitability were constructed for the three focal introduced species (*O. niloticus*, *O. esculentus* and *O. leucostictus*) using Maxent 3.3.3k. (http://www.cs.princeton.edu/~schapire/maxent/; Phillips et al., 2004, 2006). We selected linear, quadratic and hinge feature class options to avoid model overfitting, withheld 30% of data for model testing and used 10-fold cross validation of each model, and kept all other settings as default. A kernel density map of sampling effort across the region was created using the Kernel Density tool in ArcGIS v.10.5 (ESRI, Redlands, California). This was used by Maxent as a “bias file” to account for sampling bias when selecting background data. Model accuracy was measured using the area-under-curve (AUC) value of the receiver operating characteristic (ROC) curve, which ranges from 0.5 (no predictability) to 1 (perfect prediction), with values above 0.8 interpreted as a strong prediction.

## Results

### Surveys

**Fig. 2:**
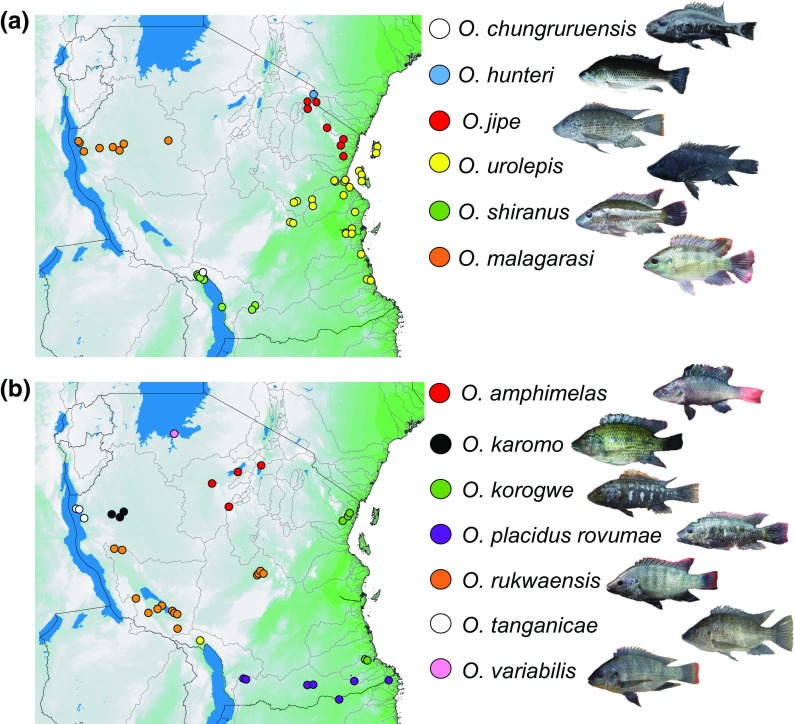
Distribution of native *Oreochromis* species across Tanzania. See Table S1 for sampling locations and coordinates. Populations within the *O*. “crater lake chambo” are not shown

In total, our data comprise 123 sites containing *Oreochromis* species, covering all major catchments in the country (Figs. 1, 2; Table 2; SI Table 1). We identified 17 *Oreochromis* taxa, of which 14 are indigenous to Tanzania and appeared to be confined to their native catchments. Two further taxa are native to Tanzania, but were translocated beyond their native range, namely *O. niloticus* (native to the Lake Tanganyika catchment), and *O. esculentus* (native to the Lake Victoria catchment). In addition, the exotic *O. leucostictus* was found to be widely distributed. Typically native *Oreochromis* tended to be restricted between one and five catchment areas (Table 2).

For most species, the distributions of native species are consistent with previous literature (Tables 1, 2), with three notable exceptions where native ranges have been reconsidered: (i) *Oreochromis korogwe* (Lowe 1955), previously known from the north of Tanzania (Pangani and Zigi river systems) was also found in south-eastern Tanzania within three lakes near Lindi (Rutamba, Nambawala and Mitupa). (ii) *Oreochromis rukwaensis* (Hilgendorf & Pappenheim 1903) previously known only from Lake Rukwa was present in an upstream section of the Ruaha river system, where a major exploited population was recorded at the Mtera Dam Lake. iii) Finally, we also observed a number of phenotypically distinct taxa in six crater lakes in the Rungwe and Kyela districts to the north of Lake Malawi. These are in addition to the previously reported *O. chungruruensis* (Ahl 1924) (Trewavas 1983). Here these six populations are nominally grouped as *O.* “crater lake chambo”.

In contrast to most native *Oreochromis*, the three introduced *Oreochromis* species were found to be widespread within Tanzania. *Oreochromis niloticus* was present at 48 of 123 sampling sites (45 translocated) and 20 of 27 catchments (19 translocated), and these included all major catchments except for the Ruvuma river and Pemba island. We noted one case where a *O. niloticus* introduction had taken place into the Upper Malagarasi region (Kazima Dam), which is in the broader Lake Tanganyika/Congo system, where *O. niloticus* is endemic. *Oreochromis esculentus* was present at 19 sampling sites (18 translocated) and 8 catchments (7 translocated), while the exotic *O. leucostictus* was present at 25 sampling sites and 9 catchments. In total, introduced species were recorded from 67 of the 123 (54.4%) sampling sites from which *Oreochromis* were recorded (Fig. 2).

### Modelling habitat preferences of introduced species

**Fig. 3:**
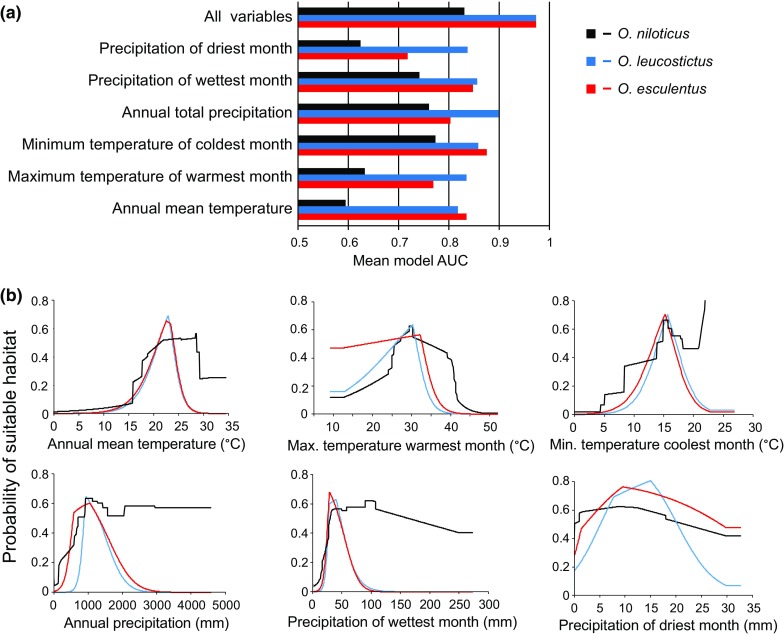
The relationship between the modelled probability of occurrence for *O. niloticus*, *O. esculentus* and *O. leucostictus* and each of the seven environmental variables included within Maxent distribution models

**Fig. 4:**
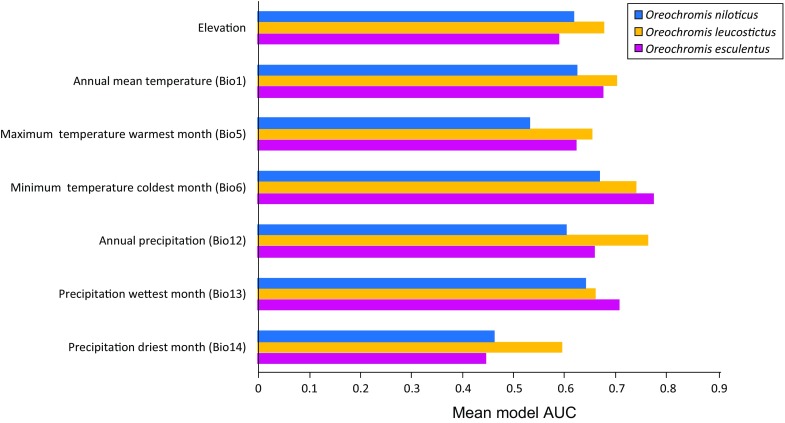
Relative predictive ability of the seven environmental variables, as measured by their AUC scores, ranging from 0 (poor fit) to 1 (perfect fit)

The Maxent models had robust evaluation metrics across replicate runs. *O. niloticus* had a mean AUC of 0.706 (standard deviation 0.063), *O. leucostictus* had a mean AUC of 0.848 (standard deviation 0.065) and *O. esculentus* had a mean AUC of 0.746 (standard deviation 0.066). Elevation, annual mean temperature, minimum temperature of the coldest month, annual precipitation and precipitation of the wettest month were consistently good predictors of distributions (Fig. 3). Response curves of species were similar, with all species having optimal habitat in elevations between 0 and 1,300 m, annual mean temperatures greater than 23°C, coldest months greater than 12°C, and annual precipitation lower than 1,300 mm per year. Notably, *O. niloticus* had the broadest thermal and elevation response curves (Fig. 4).

**Fig. 5:**
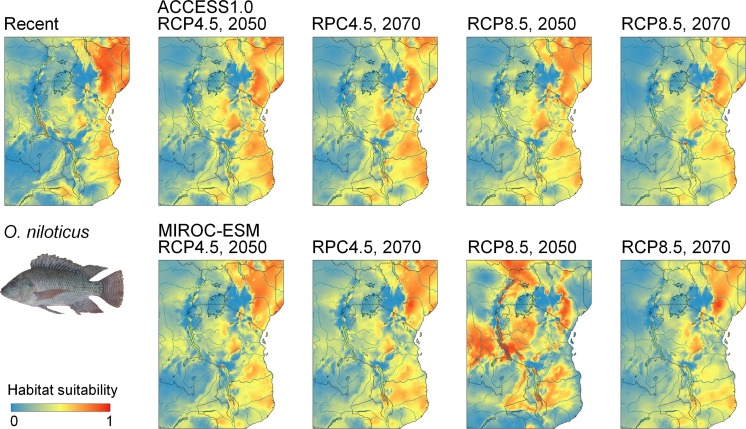
Ecological niche models of environmental suitability for *O. niloticus* in East Africa. Maps show the modelled recent and projected future distribution. Red colours represent high probability of occurrence while areas in blue are less suitable

**Fig. 6:**
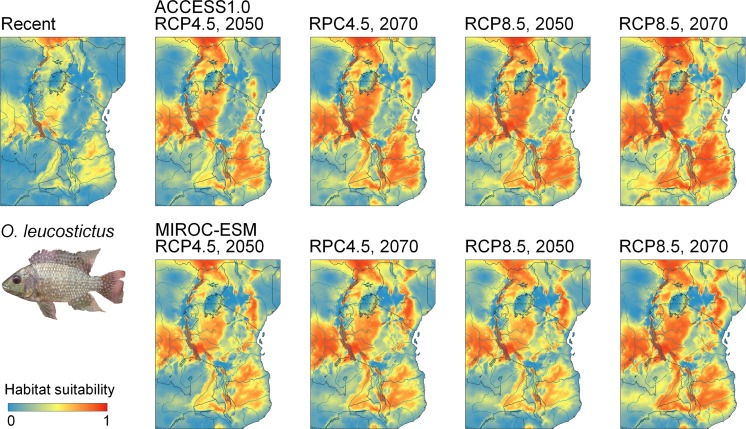
Ecological niche models of environmental suitability for *O. leucostictus* in East Africa. Maps show the modelled recent and projected future distribution. Red colours represent high probability of occurrence while areas in blue are less suitable

**Fig. 7:**
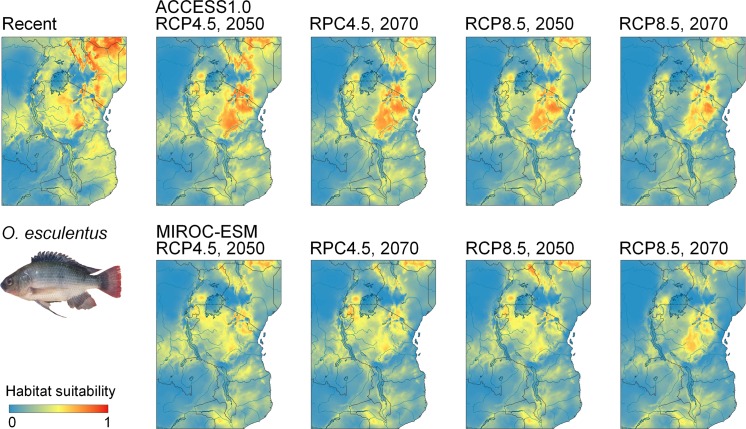
Ecological niche models of environmental suitability for *O. esculentus* in East Africa. Maps show the modelled recent and projected future distribution. Red colours represent high probability of occurrence while areas in blue are less suitable

Current suitable habitat for *O. niloticus* is widespread across East Africa, and future predicted habitat is similar to habitat that is currently suitable, with increasingly greater potential occupancy of habitat across the central region of Tanzania and other high elevation regions. The model demonstrates current habitat suitability within the Lake Malawi catchment (Fig. 5). Current habitat suitability for *O. leucostictus* is also widespread, the exception being the arid soda lake regions of central and northern Tanzania, and the high altitude Southern Highlands. Suitable habitat is projected to expand under both the RCP 4.5 and RCP 8.5 scenarios over the next 50 years, including throughout the Lake Nyasa catchment (Fig. 6). Current suitable habitat for *O. esculentus* is also broadly distributed across Tanzania, except for the high altitude and coastal regions. Suitable habitat under the RCP 4.5 and RCP 8.5 projections is projected to remain relatively unchanged (Fig. 7).

## Discussion

We have clarified the distributions of many *Oreochromis* species within Tanzania, building on local-scale work on single catchments (Lowe, 1955; Seegers, 1996), and updating previously collated information from museum collections (Trewavas, 1983; Eccles, 1992). The paucity of information on the distribution of Tanzanian tilapiine species has been highlighted in recent policy orientated work (Lind et al., 2012), and thus the core distributional information from our study should help in aquaculture planning. It will also prove useful in conservation planning and fisheries management. For example, we have been able to clarify that *O. chungruruensis* is endemic to Lake Kyungululu, whereas previous literature had used the name Lake Tschungruru (Trewavas, 1983) or incorrectly suggested the location was “probably Lake Masoko” (Eccles, 1992). Additionally, we have been able to clarify that *O. rukwaensis* supports a major fishery in the Mtera Dam lake on the Ruaha river system; previously the population has been referred to as *O. urolepis* (Mwalyosi 1986; Chale 2004). Although *O. urolepis* is commonplace from the Kidatu Dam and further downstream on the Ruaha system, we have not encountered *O. urolepis* in the Mtera Dam, or any site further upstream. Previously, *O. rukwaensis* was regarded as endemic to the neighbouring Lake Rukwa catchment (Eccles, 1992; Trewavas, 1983), and it appears likely that upper Ruaha population is native, although this interpretation requires additional evidence from a population genetic study of the species.

### Native species

The findings of our surveys have confirmed the results of earlier studies reporting distributions of many of the native species within Tanzania, and support the information used in conservation assessments for the IUCN Red List of Threatened Species, and associated summary documents (Darwall et al., 2005). The status of one possible native species record remains unresolved. There is a report of *Oreochromis spilurus* (Günther 1894) in the Momella lakes of Arusha National Park (Trewavas, 1983), which would represent the southern range limit of the species. These lakes were not sampled during our survey, but specimens possibly corresponding to this species have been previously collected by one author (B.P. Ngatunga) from Lake Longil near the Momella lakes in 2002.

Species with the narrowest distributions are the IUCN listed Critically Endangered crater lake endemics, namely *Oreochromis hunteri* Günther 1889 in Lake Chala and *O. chungruruensis* from Lake Kyungululu (Trewavas 1983). Lake Kyungululu is one of a series of crater lakes in the Kyela and Rungwe districts, and in six other lakes we found populations of *Oreochromis* bearing pigmentation patterns resembling species from the Lake Malawi “chambo” group, namely *Oreochromis squamipinnis* (Günther, 1864) and *Oreochromis karongae* (Trewavas, 1941). Further work is needed to establish the evolutionary affinities of these populations, so here we retain them in the general grouping *O*. “crater lake chambo”. It is plausible that they represent either allopatric variants of Lake Malawi species, or possibly natural hybrids. Previous research on this crater lake system has suggested that the Lake Malawi catchment endemic *Oreochromis lidole* (Trewavas, 1941) is present in two lakes, Lake Kyungululu (= Chungruru) and Lake Kingiri (Trewavas, 1983); however, we did not encounter this species during our sampling. It is possible with more intensive sampling of these locations, and others, that further rarer species will be found.

Our findings are consistent with several species having very restricted distributions within catchments, despite an absence of clear geographical barriers to wider dispersal. These include *O. variabilis* (Boulenger 1906), a species recorded on the IUCN Red List as Critically Endangered. It is now almost entirely extirpated from its native range in the Lake Victoria catchment following introductions of Nile perch *Lates niloticus* (L. 1758), *O. niloticus* and *O. leucostictus* from the 1950s onwards. To our knowledge, these records are the first reported observations of the population at Makobe Island in Lake Victoria since the 1990s (Seehausen, 1996). *Oreochromis variabilis* has only otherwise been reported within the last 15 years from one location in Lake Victoria (Oele Beach in Kenya; Maithya et al., 2012), and several satellite water bodies, including Lakes Burigi, Ikimba, Katwe and Kubigena in Tanzania (Katunzi & Kishe, 2004) and the Mamboleo, Komondi and Kalenjouk Dams in Kenya (Maithya et al., 2012). The species was also trialled in aquaculture ponds in the 1950s in Korogwe in the Pangani system (Lowe-McConnell, 2006), but was not encountered in the Pangani during our sampling. Other species with restricted distributions in single catchments include *O. karomo* (Poll, 1948), another species listed by the IUCN Red List as Critically Endangered, which we found at three of our sampling sites in the upper reaches of the Malagarasi river system.

Our study has extended the known distributions of three species, in addition to the range extension of *O. rukwaensis*. In the north of Tanzania, *O. jipe* has only been formally recorded from Lake Jipe and Nyumba ya Mungu, and this narrow distribution has contributed to an IUCN Red List assessment of Critically Endangered. Lowe (1955) originally described four new species from the Pangani system: *O. korogwe, O. jipe*, *Oreochromis girigan* (Lowe 1955) and *Oreochromis pangani* (Lowe 1955). However, it has been suggested that the last three are conspecific (Seegers et al., 2003; Seegers, 2008), and with page priority, the correct name would be *O. jipe*, as listed by Eschmeyer (2017). We could find no obvious basis for distinguishing more than a single species from this group, and so we consider that our sampling indicates that *O. jipe* is widespread throughout the Pangani system, including water bodies peripheral to the main channel, such as Lake Kalimau.

In the Lower Pangani system, we found *O. jipe* co-occurring with *O. korogwe*, a species originally described using a collection made from government experimental aquaculture ponds in Korogwe (Lowe, 1955). Subsequently, the natural distribution was reported to extend to coastal stretches of the Pangani and neighbouring Zigi rivers, and it has also been introduced to the Mlingano Dam near Tanga (Trewavas, 1983). Our sampling confirmed this distribution in the north of Tanzania. There are additional reports of *O. korogwe* (Dieleman et al., 2015) and *O. pangani* (now *O. jipe*) (Dadzie et al., 2000) from Lake Chala. From our observations of samples collected at Lake Chala, we could not confirm these records, and the identity of a second sympatric species reported by Dieleman et al. (2015) in the crater lake requires clarification. Our study has, however, confirmed that *O. korogwe* has a distribution broader than reported by Trewavas (1983). We found it to be present in three lakes near Rutamba in southern Tanzania. The population in Lake Rutamba had previously been sampled in 1982, but the few small specimens collected were assigned to *Oreochromis placidus* (Trewavas 1941) by Trewavas (1983). With the benefit of a large collection of freshly collected specimens, the characteristic checkered patterned of the females and immature males can be seen, along with the diagnostic pale flank bars of sexually mature male *O. korogwe*. We did not record *O. placidus* outside of the Ruvuma and Lukuledi river systems, both of which are well to the south of the Rutamba lakes. Furthermore, we were unable to identify any clear phenotypic differences between specimens of *O. placidus* and *O. shiranus*. Previous studies have made no effort to provide features that distinguish among these taxa [e.g. Eccles (1992), Trewavas (1983)] and we suspect that they are best considered conspecific, in which event *O. shiranus* would be the senior synonym. However, we have provisionally retained the species distinction here according to catchment of occupancy until these can be further investigated.

Finally, we also collected *Oreochromis amphimelas* (Hilgendorf, 1905) from Lake Sulungali (often labelled as Lake Sulunga on maps) near Dodoma therefore extending its range. This is a large shallow endorheic lake prone to fluctuations in salinity associated with water level changes, presenting similar conditions to the known localities for this species in Lakes Manyara, Eyasi, Singida and Kitangiri (Eccles, 1992). At present it is unclear if this *O. amphimelas* has been introduced to Lake Sulungali or is native to the catchment.

### Introduced species

The most striking results of the survey are the broad distributions of three introduced species across Tanzania. The Nile tilapia (*O. niloticus*) is native to Tanzania, and has a natural distribution within the Lake Tanganyika catchment, where it is relatively uncommon and largely confined to river mouths (Trewavas 1983; Kullender & Roberts 2011). We recorded *O. niloticus* in all major basins. The widespread distribution of the species appears to be largely a consequence of deliberate stocking of water bodies in attempts to improve fishery production, although feral populations may also be present following escapes from aquaculture facilities. The earliest introductions of *O. niloticus* into Lake Victoria took place during the 1950s (Goudswaard et al., 2002) and were sourced from elsewhere in Nile catchment, potentially Lake Edward (Mwanja et al., 2008). Interestingly, the native Lake Tanganyika population of Nile tilapia does not seem to have been widely stocked, and instead the introduced Lake Victoria population is generally cited by local officials as the source of stocks that have been translocated across Tanzania; however, it is plausible that some of the introductions were from other sources. Recently, in 2016, the Chitralada strain of *O. niloticus* variety has been imported from Thailand to ponds in Dar-es-Salaam (Shechonge & Ngatunga, pers. obs.)

The blue-spotted tilapia (*O. leucostictus*) is naturally distributed in southerly reaches of the Nile system, including Lakes Edward, Albert and George. The first recorded observations of the species in Tanzania were within Lake Victoria, where it was probably introduced alongside *O. niloticus* and *Coptodon zillii* (Gervais 1848) during the 1950s (Goudswaard et al., 2002). To our knowledge, the species had not previously been recognised from any Tanzanian habitat outside the Lake Victoria system, except one location in the Lake Malawi catchment where it was reported from a survey in 2011 (Genner et al., 2013). The species is relatively small bodied (23.2 cm maximum SL; Table 1) compared to Nile tilapia (60.0 cm maximum SL; Table 1), and is typically found in shallow vegetated habitats (Lowe-McConnell, 2006). The co-distribution of *O. leucostictus* with *O. niloticus* across Tanzania is suggestive of *O. leucostictus* stock being misidentified as the favoured *O. niloticus*: we have found mixtures of the species at two hatcheries that have supplied fingerlings (labelled as *O. niloticus*) to many fish farmers. It is plausible that species may hybridise (Nyingi & Agnèse, 2007), which requires further investigation. It is clear that the species has a strong ability to spread throughout river systems, exemplified by the widespread and previously unreported distribution of the species across most of the sites we sampled within the Malagarasi system, from shallow swampy lakes, to the main river channel and the peripheral swampy habitats of Lake Tanganyika.

The Singida tilapia (*Oreochromis esculentus*) is endemic to the Lake Victoria basin, where it has been largely extirpated from the system, and has not been recorded from the main water body for many years. Within the last 15 years, it has been reported from several satellite lakes of Lake Victoria within the Tanzania sector of the catchment, including Lake Burigi, Lake Ikimba, Lake Katwe and Lake Kirumi (Katunzi & Kishe, 2004). We found *O. esculentus* in Lake Malimbe in 2016, updating observations by Katunzi and Kishe, who also reported it as present. The species was introduced into several other catchments in Tanzania during the 1950s, and our surveys confirm their continued presence. We found *O. esculentus* in the Pangani basin including Lake Jipe, lakes in the central regions (Lake Kitangiri and Lake Hombolo) and also Lake Rukwa in the southwest of the country. In many of these lakes, the species comprises a significant part of the fishery production (A. Shechonge, M. Genner, BP. Ngatunga and G. Turner pers obs.). Our study has also extended the known distribution of *O. esculentus* to the upper reaches of the Malagarasi system.

Our modelling results showed that while all three species that have been introduced beyond their native ranges had similar ecological tolerances, *O. esculentus* and *O. leucostictus* were relatively conservative in their habitat use patterns, relative to *O. niloticus*. This could be suggestive of *O. niloticus* having broader natural ecological tolerances than the other non-native species; however, current distributional ranges do not always fully reflect ecological tolerances of species (Bosci et al., 2016). Our forward predictions suggest that the potential spread of all these species over the next 50 years is unlikely to be significantly limited by a lack of suitable habitat. Ultimately, the likelihood of establishment beyond the current range of these species will depend on the extent of further human introductions into new catchments, in addition to the ability of species to disperse and establish within the river systems that they currently occupy. It is plausible that all species could experience rapid selection that enable them to tolerate broader climatic conditions. Additionally, it is important to consider the limitation of a species distribution modelling approach. Here we used only atmospheric variables in the predictive model, and did not consider aquatic environmental variables, or interactions with other species. We also focussed on only two readily accessible sets of global climate models for each of the scenarios and did not consider variation from multiple realisations within a climate model. Plausibly, use of a broader range of models and realisations would provide greater accuracy (Porfirio et al., 2014).

There are records of other *Oreochromis* being introduced to non-native locations around Tanzania that we did not encounter during surveys. *Oreochromis macrochir* (Boulenger, 1912), naturally distributed in the Zambezi and neighbouring systems, was reportedly introduced to aquaculture ponds the Pangani system (Dadzie et al., 2000). *Oreochromis mossambicus* (Peters, 1852), naturally distributed in coastal rivers from the Zambezi to Bushman river systems of south-eastern Africa, has also been listed as invasive in Tanzania by The Centre for Agriculture and Bioscience International (CABI) Invasive Species Compendium (http://www.cabi.org/). We did not confirm the presence of this species at any site in Tanzania, but note that many local field workers seem to readily misidentify sexually mature males of native species, such as *O. urolepis* and *O. placidus,* as *O. mossambicus*. Finally, *Oreochromis variabilis* was historically reported from aquaculture ponds in the Pangani system (Dadzie et al., 2000; Lowe-McConnell, 2006). It is plausible that further sampling in these regions, including increased effort in the locations we sampled, will yield further *Oreochromis* diversity.

### Distributions and conservation

The impacts of introduced *Oreochromis* species on native components of the fish communities in Tanzania are currently unclear. In principle, negative impacts could include competition for limited resources, predation upon eggs and juveniles, enhanced spread of parasites and pathogens and hybridisation with native species. The majority of work on invasive species in East Africa has been focussed on Lake Victoria, where the decline of the endemic tilapiine and haplochromine faunas coincided with the introduction of the Nile perch, Nile tilapia and the redbelly tilapia (*Coptodon zillii*) (Ogutu-Ohwayo, 1990; Balirwa, 1992). Direct evidence of predation by Nile perch on the haplochromines provided strong evidence for a role of this species in the extinction of many species (Kishe-Machumu et al., 2012), but the impact of the tilapiines on the native species is still largely unclear. This is partly due to the many other changes taking place in the system over the same timescale, including widespread eutrophication and extensive fisheries operations (Verschuren et al., 2002; Hecky et al., 2010). Field surveys and experimental manipulations are required to more fully understand the ecological impact of these species in Tanzania, particularly in light of the negative ecological impacts that *O. niloticus* has had in other parts of its introduced range (Canonico et al., 2005).

Evidence of hybridisation among native and non-native species is however more widespread. Hybridisation of *O. niloticus* with native species has been established in many species in Africa, including *O. mossambicus* in southern Africa (Firmat et al. 2013), *Oreochromis andersonii* (Castelnau, 1861) and *O. macrochir* in Zambia (Deines et al. 2014) and *O. esculentus* in satellite lakes of Lake Victoria (Mwanja & Kaufman, 1995; Angienda et al., 2011, Mwanja et al., 2012; but see Agnése et al., 1999). Additionally, hybrids between *O. leucostictus* and *O. niloticus* have been identified in Kenya (Nyingi & Agnèse, 2007; Ndiwa et al., 2014), and hybrids between *O. esculentus* and *O. amphimelas* are reported from Lake Kitangiri in Tanzania (Trewavas & Fryer, 1965). It is therefore plausible that hybridisation among stocked and native *Oreochromis* species is taking place in Tanzania, but the extent of this is yet to be determined. Given the declining cost of genome sequencing, and the recent publication of the *Oreochromis niloticus* genomic resources (Brawand et al., 2014), genome-wide evidence has great potential to uncover patterns of population structure and genetic admixture among these species.

### Zoned aquaculture and capture fisheries development

Global aquaculture production was an estimated 73.8 million tonnes in 2014 (FAO, 2016), with inland freshwater facilities making up the majority with 47.1 million tonnes. Increasingly, tilapiine cichlid species are important contributors to this inland production comprising ~ 3.5 million tonnes in 2010, with Asia being largest producer (Bostock et al., 2010; FAO, 2016). With the combination of an increased reliance of fish protein, and the projected global population expansion to 9.7 billion people by 2050, it has been estimated that fish demand from aquaculture will more than double to 100 million tonnes by 2025 (FAO, 2016), and 60% of this increase will comprise freshwater species including carps, *Pangasius* and Nile tilapia (FAO, 2016). Currently Africa produces only 2.3% of global aquaculture biomass (FAO, 2016), and there is increasing recognition that there will be considerable development of aquaculture industry across the continent in the coming decades. This will be essential to meet the increasing supply gap between capture fisheries production and demand for fish protein (Edwards, 2015). Given this background, the expansion of tilapiine-based aquaculture in Africa is very likely.

Our results demonstrate that aquaculture development based on tilapiine species that are not native to catchments is widespread in Tanzania. However, an alternative approach is to utilise large-bodied species that are native to the catchments where aquaculture facilities are established (Lind et al., 2012). This “zoned aquaculture” approach provides assurance that escapes will not lead to substantial environmental impacts for native species, but also have potential commercial benefits. These include production of fish that have established markets, and the ready access of hatcheries to wild genetic resources for inclusion in breeding stock. This is particularly important, given evidence that stocks in tilapia aquaculture systems in Africa rapidly become inbred and lose desirable traits such as large growth because small bodied and early maturation are favoured by selection in aquaculture systems, a problem exacerbated when non-native strains are introduced via a small number of founders (Brummett et al., 2004). Furthermore, uncontrolled movements of species among catchments increase the risk of introduction of lethal infections such as Tilapia Lake Virus (Eyngor et al., 2014). Our study provides strong evidence that native large-bodied species are present in all major catchments of Tanzania that we suggest may be tested for suitability for pond and cage aquaculture through the use of controlled experiments. Finally, although farmed tilapias have been widely stocked into natural waterbodies and reservoirs in Tanzania, almost without exception these already contained native tilapia species. Ideally, if stocking of invasive species is to continue, we require evidence that stocking of tilapias can enhance the fishery production given the particular ecological circumstances. Perhaps the best evidence that it can develop fisheries in some situations comes from the introduction of specialised offshore lake-living *O. esculentus* to exploit offshore niches in large lakes and reservoirs. The least likely cases of stocking helping to increase biomass production come from the recent widespread stocking of the invasive, inshore-specialist, small-maturing *O. leucostictus*.

To conclude, here we report the widespread distribution of non-native *Oreochromis* species in Tanzania. Further work is needed to establish the distributions of other tilapiine species within the country, including *Coptodon zillii* and *Coptodon rendalli* (Boulenger 1897). Moreover, during our work we have not attributed introductions to specific causes (aquaculture or capture fisheries development), and further work is needed to fully understand the relative roles of these in generating the patterns observed. Escapes from aquaculture facilities can lead to establishment of populations in the wild, for example, we observed *O. leucostictus* in a river geographically proximate to aquaculture ponds in the Lake Rukwa catchment (Supplementary Information 1). This suggests that future work may be able to predict the likelihood of invasion of the natural habitat using proxies related to the intensity of the aquaculture in a region.

## Electronic supplementary material

Below is the link to the electronic supplementary material.

Supplementary material 1 (DOCX 66 kb)
