# Supplementary material for: Widespread colonisation of Tanzanian catchments by introduced Oreochromis tilapia fishes: the legacy from decades of deliberate introduction
Source: Hydrobiologia. 2018 Apr 4;832(1):235–53. doi: 10.1007/s10750-018-3597-9 (PMC6394791; doi:10.1007/s10750-018-3597-9)
Supplement: Supplementary file 1 — Supplementary material 1 (DOCX 66 kb) [file 10750_2018_3597_MOESM1_ESM.docx]

**Supplementary Information 1:** Collection Localities of *Oreochromis* in Tanzania 2011-2017.

| **Year** | **Month** | **Day** | **Site Code** | **Latitude** | **Longitude** | **Site** | **Location name** | **Catchment** | O. esculentus | O. leucostictus | O. niloticus | O. placidus | O. rukwaensis | O. shiranus | O. urolepis | O. jipe (complex) | O. amphimelas | O. korogwe | O. variabilis | O. chungruruensis | O. karomo | O. tanganicae | O. malagarasi | O. hunteri | O. "Crater lake chambo" |
| --- | --- | --- | --- | --- | --- | --- | --- | --- | --- | --- | --- | --- | --- | --- | --- | --- | --- | --- | --- | --- | --- | --- | --- | --- | --- |
| 2011 | 7 | 14 | 14_07_11 #2 | -9.551 | 33.887 | 1 | Pool near Kingiri | Lake Malawi | 0 | 0 | 0 | 0 | 0 | 1 | 0 | 0 | 0 | 0 | 0 | 0 | 0 | 0 | 0 | 0 | 0 |
| 2011 | 7 | 14 | 14_07_11 #1 | -9.412 | 33.857 | 2 | Lake Kingiri | Lake Malawi | 0 | 0 | 0 | 0 | 0 | 1 | 0 | 0 | 0 | 0 | 0 | 0 | 0 | 0 | 0 | 0 | 1 |
| 2011 | 7 | 15 | 15_07_11 #3 | -9.393 | 33.830 | 3 | Lake Ilamba | Lake Malawi | 0 | 0 | 0 | 0 | 0 | 1 | 0 | 0 | 0 | 0 | 0 | 0 | 0 | 0 | 0 | 0 | 1 |
| 2011 | 7 | 17 | 17_07_11 #5 | -9.334 | 33.756 | 4 | Lake Massoko | Lake Malawi | 0 | 0 | 0 | 0 | 0 | 0 | 0 | 0 | 0 | 0 | 0 | 0 | 0 | 0 | 0 | 0 | 1 |
| 2011 | 7 | 18 | 18_07_11 #7 | -9.308 | 33.865 | 5 | Lake Kyungululu | Lake Malawi | 0 | 0 | 0 | 0 | 0 | 0 | 0 | 0 | 0 | 0 | 0 | 1 | 0 | 0 | 0 | 0 | 0 |
| 2011 | 7 | 20 | 20_07_11 #11 | -9.537 | 33.887 | 6 | Pool near Kyela | Lake Malawi | 0 | 0 | 0 | 0 | 0 | 1 | 0 | 0 | 0 | 0 | 0 | 0 | 0 | 0 | 0 | 0 | 0 |
| 2011 | 7 | 20 | 20_07_11 #9 | -9.367 | 33.808 | 7 | Lake Ikapu | Lake Malawi | 0 | 0 | 0 | 0 | 0 | 1 | 0 | 0 | 0 | 0 | 0 | 0 | 0 | 0 | 0 | 0 | 1 |
| 2011 | 11 | 22 | 22_11_11_Itamba | -9.353 | 33.843 | 8 | Lake Itamba | Lake Malawi | 0 | 0 | 1 | 0 | 0 | 0 | 0 | 0 | 0 | 0 | 0 | 0 | 0 | 0 | 0 | 0 | 1 |
| 2011 | 11 | 27 | 27_11_11_Itende | -9.322 | 33.788 | 9 | Lake Itende | Lake Malawi | 0 | 0 | 0 | 0 | 0 | 0 | 0 | 0 | 0 | 0 | 0 | 0 | 0 | 0 | 0 | 0 | 1 |
| 2011 | 12 | 1 | 01_12_11 #R4 | -7.466 | 36.517 | 10 | Ruaha river | Ruaha / Rufiji | 0 | 0 | 1 | 0 | 0 | 0 | 0 | 0 | 0 | 0 | 0 | 0 | 0 | 0 | 0 | 0 | 0 |
| 2011 | 12 | 2 | 02_12_11_Ruvu | -6.698 | 38.703 | 11 | Ruvu river | Ruvu river | 0 | 0 | 0 | 0 | 0 | 0 | 1 | 0 | 0 | 0 | 0 | 0 | 0 | 0 | 0 | 0 | 0 |
| 2012 | 9 | 2 | 02_09_12 #4 | -8.409 | 32.926 | 12 | Lake Rukwa | Lake Rukwa | 1 | 0 | 0 | 0 | 1 | 0 | 0 | 0 | 0 | 0 | 0 | 0 | 0 | 0 | 0 | 0 | 0 |
| 2012 | 9 | 3 | 03_09_12 #5 | -8.397 | 32.902 | 13 | Lake Rukwa | Lake Rukwa | 0 | 0 | 0 | 0 | 1 | 0 | 0 | 0 | 0 | 0 | 0 | 0 | 0 | 0 | 0 | 0 | 0 |
| 2012 | 9 | 5 | 05_09_12 #10 | -8.708 | 34.388 | 14 | Rujewa ponds | Ruaha / Rufiji | 0 | 0 | 1 | 0 | 0 | 0 | 0 | 0 | 0 | 0 | 0 | 0 | 0 | 0 | 0 | 0 | 0 |
| 2012 | 9 | 6 | 06_09_12 #11 | -10.710 | 35.199 | 15 | Kitai prison dam | Ruvuma | 0 | 0 | 0 | 1 | 0 | 0 | 0 | 0 | 0 | 0 | 0 | 0 | 0 | 0 | 0 | 0 | 0 |
| 2012 | 9 | 6 | 06_09_12 #12 | -10.707 | 35.248 | 16 | Liganga river | Ruvuma | 0 | 0 | 0 | 1 | 0 | 0 | 0 | 0 | 0 | 0 | 0 | 0 | 0 | 0 | 0 | 0 | 0 |
| 2012 | 9 | 6 | 06_09_12 #14 | -8.443 | 33.013 | 17 | Mkwajuni market (samples from Lake Rukwa) | Lake Rukwa | 1 | 0 | 0 | 0 | 1 | 0 | 0 | 0 | 0 | 0 | 0 | 0 | 0 | 0 | 0 | 0 | 0 |
| 2012 | 9 | 9 | 09_09_12 #19 | -10.502 | 35.671 | 18 | Lumecha river (Ruhuhu tributory) | Lake Malawi | 0 | 0 | 0 | 0 | 0 | 1 | 0 | 0 | 0 | 0 | 0 | 0 | 0 | 0 | 0 | 0 | 0 |
| 2012 | 9 | 9 | 09_09_12 #18 | -7.064 | 35.853 | 19 | Songwa market (samples from Mtera dam) | Ruaha / Rufiji | 0 | 0 | 0 | 0 | 1 | 0 | 0 | 0 | 0 | 0 | 0 | 0 | 0 | 0 | 0 | 0 | 0 |
| 2012 | 9 | 10 | 10_09_12 #21 | -10.623 | 35.636 | 20 | Songea ponds (Ruhuhu tributory) | Lake Malawi | 0 | 1 | 1 | 0 | 0 | 0 | 0 | 0 | 0 | 0 | 0 | 0 | 0 | 0 | 0 | 0 | 0 |
| 2013 | 8 | 13 | 13_08_13 #3 | -8.719 | 39.265 | 21 | Miteja | Miteja | 0 | 0 | 0 | 0 | 0 | 0 | 1 | 0 | 0 | 0 | 0 | 0 | 0 | 0 | 0 | 0 | 0 |
| 2013 | 8 | 13 | 13_08_13 #2 | -8.010 | 38.968 | 22 | Mkapa bridge, Rufiji river | Ruaha / Rufiji | 0 | 0 | 0 | 0 | 0 | 0 | 1 | 0 | 0 | 0 | 0 | 0 | 0 | 0 | 0 | 0 | 0 |
| 2013 | 8 | 14 | 14_08_13 #7 | -10.032 | 39.460 | 23 | Lake Rutamba | Rutamba lakes | 0 | 0 | 1 | 0 | 0 | 0 | 0 | 0 | 0 | 1 | 0 | 0 | 0 | 0 | 0 | 0 | 0 |
| 2013 | 8 | 14 | 14_08_13 #6 | -9.576 | 39.496 | 24 | Mbwenkuru river (market nearby) | Mbwenkuru | 0 | 0 | 0 | 0 | 0 | 0 | 1 | 0 | 0 | 0 | 0 | 0 | 0 | 0 | 0 | 0 | 0 |
| 2013 | 8 | 15 | 15_08_13 #12 | -10.567 | 38.926 | 25 | Nambuti river | Lukuledi | 0 | 0 | 0 | 1 | 0 | 0 | 0 | 0 | 0 | 0 | 0 | 0 | 0 | 0 | 0 | 0 | 0 |
| 2013 | 8 | 15 | 15_08_13 #10 | -10.382 | 39.204 | 26 | Roadside pool | Lukuledi | 0 | 0 | 1 | 0 | 0 | 0 | 0 | 0 | 0 | 0 | 0 | 0 | 0 | 0 | 0 | 0 | 0 |
| 2013 | 8 | 15 | 15_08_13 #13 | -10.567 | 38.925 | 27 | Lukuledi tributory | Lukuledi | 0 | 0 | 0 | 1 | 0 | 0 | 0 | 0 | 0 | 0 | 0 | 0 | 0 | 0 | 0 | 0 | 0 |
| 2013 | 8 | 16 | 16_08_13 #15 | -11.414 | 38.492 | 28 | Ruvuma river | Ruvuma | 0 | 0 | 0 | 1 | 0 | 0 | 0 | 0 | 0 | 0 | 0 | 0 | 0 | 0 | 0 | 0 | 0 |
| 2013 | 8 | 17 | 17_08_13 #19 | -10.869 | 37.641 | 29 | Namiungo river | Ruvuma | 0 | 0 | 0 | 1 | 0 | 0 | 0 | 0 | 0 | 0 | 0 | 0 | 0 | 0 | 0 | 0 | 0 |
| 2013 | 8 | 17 | 17_08_13 #18 | -10.847 | 37.474 | 30 | Muhuwesi river | Ruvuma | 0 | 0 | 0 | 1 | 0 | 0 | 0 | 0 | 0 | 0 | 0 | 0 | 0 | 0 | 0 | 0 | 0 |
| 2013 | 8 | 18 | 18_08_13 #20 | -10.597 | 40.155 | 31 | Lake Chidya | Ruvuma | 0 | 0 | 0 | 1 | 0 | 0 | 0 | 0 | 0 | 0 | 0 | 0 | 0 | 0 | 0 | 0 | 0 |

**Supplementary Information 1: continued**

| **Year** | **Month** | **Day** | **Site Code** | **Latitude** | **Longitude** | **Site** | **Location name** | **Catchment** | O. esculentus | O. leucostictus | O. niloticus | O. placidus | O. rukwaensis | O. shiranus | O. urolepis | O. jipe (complex) | O. amphimelas | O. korogwe | O. variabilis | O. chungruruensis | O. karomo | O. tanganicae | O. malagarasi | O. hunteri | O. "Crater lake chambo" |
| --- | --- | --- | --- | --- | --- | --- | --- | --- | --- | --- | --- | --- | --- | --- | --- | --- | --- | --- | --- | --- | --- | --- | --- | --- | --- |
| 2013 | 8 | 20 | 20_08_13 #21 | -10.360 | 39.776 | 32 | Kitele Lake | Kitele lake | 0 | 0 | 1 | 0 | 0 | 0 | 0 | 0 | 0 | 0 | 0 | 0 | 0 | 0 | 0 | 0 | 0 |
| 2013 | 8 | 20 | 20_08_13 #24 | -8.006 | 38.769 | 33 | Chemi chemi hot springs lake | Ruaha / Rufiji | 0 | 0 | 0 | 0 | 0 | 0 | 1 | 0 | 0 | 0 | 0 | 0 | 0 | 0 | 0 | 0 | 0 |
| 2013 | 8 | 20 | 20_08_13 #23 | -7.994 | 38.759 | 34 | Utete market (main Rufiji channel) | Ruaha / Rufiji | 0 | 0 | 0 | 0 | 0 | 0 | 1 | 0 | 0 | 0 | 0 | 0 | 0 | 0 | 0 | 0 | 0 |
| 2013 | 8 | 20 | 20_08_13 #22 | -7.991 | 38.749 | 35 | Oxbow lake, near Utete | Ruaha / Rufiji | 0 | 0 | 0 | 0 | 0 | 0 | 1 | 0 | 0 | 0 | 0 | 0 | 0 | 0 | 0 | 0 | 0 |
| 2013 | 8 | 20 | 20_08_13 #25 | -7.858 | 38.962 | 36 | Luhiho river | Ruaha / Rufiji | 0 | 0 | 0 | 0 | 0 | 0 | 1 | 0 | 0 | 0 | 0 | 0 | 0 | 0 | 0 | 0 | 0 |
| 2014 | 1 | 22 | 22_01_14 #2 | -6.246 | 38.388 | 37 | Wami river | Wami river | 0 | 0 | 0 | 0 | 0 | 0 | 1 | 0 | 0 | 0 | 0 | 0 | 0 | 0 | 0 | 0 | 0 |
| 2014 | 1 | 22 | 22_01_14 #1 | -6.242 | 38.380 | 38 | Wami river | Wami river | 0 | 0 | 0 | 0 | 0 | 0 | 1 | 0 | 0 | 0 | 0 | 0 | 0 | 0 | 0 | 0 | 0 |
| 2014 | 1 | 23 | 23_01_14 #4 | -6.247 | 38.688 | 39 | Matipwili Pond (Wami river) | Wami river | 0 | 0 | 0 | 0 | 0 | 0 | 1 | 0 | 0 | 0 | 0 | 0 | 0 | 0 | 0 | 0 | 0 |
| 2014 | 1 | 23 | 23_01_14 #3 | -6.240 | 38.707 | 40 | Wami river | Wami river | 0 | 0 | 0 | 0 | 0 | 0 | 1 | 0 | 0 | 0 | 0 | 0 | 0 | 0 | 0 | 0 | 0 |
| 2014 | 1 | 25 | 25_01_14 #5 | -7.118 | 35.963 | 41 | Mtera Dam (Ruaha) | Ruaha / Rufiji | 0 | 0 | 0 | 0 | 1 | 0 | 0 | 0 | 0 | 0 | 0 | 0 | 0 | 0 | 0 | 0 | 0 |
| 2014 | 1 | 26 | 26_01_14 #6 | -7.105 | 35.827 | 42 | Mtera Dam (Ruaha) | Ruaha / Rufiji | 0 | 0 | 0 | 0 | 1 | 0 | 0 | 0 | 0 | 0 | 0 | 0 | 0 | 0 | 0 | 0 | 0 |
| 2014 | 1 | 28 | 28_01_14 #10 | -5.952 | 35.943 | 43 | Hombolo Dam (Wami) | Wami river | 1 | 0 | 0 | 0 | 0 | 0 | 0 | 0 | 0 | 0 | 0 | 0 | 0 | 0 | 0 | 0 | 0 |
| 2014 | 1 | 28 | 28_01_14 #9 | -5.951 | 35.969 | 44 | Hombolo Dam (Wami) | Wami river | 1 | 0 | 1 | 0 | 0 | 0 | 0 | 0 | 0 | 0 | 0 | 0 | 0 | 0 | 0 | 0 | 0 |
| 2014 | 1 | 29 | 29_01_14 #12 | -7.661 | 36.978 | 45 | Great Ruaha near Kidatu | Ruaha / Rufiji | 0 | 0 | 0 | 0 | 0 | 0 | 1 | 0 | 0 | 0 | 0 | 0 | 0 | 0 | 0 | 0 | 0 |
| 2014 | 1 | 29 | 29_01_14 #11 | -6.865 | 37.608 | 46 | Mindu Dam (Ruvu) | Ruvu river | 0 | 1 | 1 | 0 | 0 | 0 | 1 | 0 | 0 | 0 | 0 | 0 | 0 | 0 | 0 | 0 | 0 |
| 2014 | 11 | 5 | 05_11_14 #1 | -3.317 | 37.696 | 47 | Lake Chala | Lake Chala | 0 | 0 | 0 | 0 | 0 | 0 | 0 | 0 | 0 | 0 | 0 | 0 | 0 | 0 | 0 | 1 | 0 |
| 2015 | 1 | 22 | 22_01_15 #1 | -4.060 | 34.194 | 48 | Lake Kitangiri | Lake Eyasi | 0 | 1 | 1 | 0 | 0 | 0 | 0 | 0 | 1 | 0 | 0 | 0 | 0 | 0 | 0 | 0 | 0 |
| 2015 | 1 | 23 | 23_01_15 #2 | -4.794 | 34.751 | 49 | Lake Singida | Lake Singida | 0 | 0 | 1 | 0 | 0 | 0 | 0 | 0 | 1 | 0 | 0 | 0 | 0 | 0 | 0 | 0 | 0 |
| 2015 | 1 | 23 | 23_01_15 #3 | -4.377 | 35.085 | 50 | Lake Basotu | Lake Basotu | 1 | 0 | 0 | 0 | 0 | 0 | 0 | 0 | 0 | 0 | 0 | 0 | 0 | 0 | 0 | 0 | 0 |
| 2015 | 1 | 24 | 24_01_15 #4 | -3.632 | 35.068 | 51 | Lake Eyasi | Lake Eyasi | 0 | 0 | 1 | 0 | 0 | 0 | 0 | 0 | 1 | 0 | 0 | 0 | 0 | 0 | 0 | 0 | 0 |
| 2015 | 1 | 25 | 25_01_15 #5 | -3.877 | 35.882 | 52 | Lake Burunge | Lake Burugi | 0 | 0 | 1 | 0 | 0 | 0 | 0 | 0 | 0 | 0 | 0 | 0 | 0 | 0 | 0 | 0 | 0 |
| 2015 | 1 | 25 | 25_01_15 #6 | -3.622 | 35.806 | 53 | Lake Manyara | Lake Manyara | 0 | 0 | 1 | 0 | 0 | 0 | 0 | 0 | 0 | 0 | 0 | 0 | 0 | 0 | 0 | 0 | 0 |
| 2015 | 3 | 10 | 10_03_15 #1 | -7.276 | 39.086 | 54 | Lake Mansi | Lake Mansi | 0 | 0 | 0 | 0 | 0 | 0 | 1 | 0 | 0 | 0 | 0 | 0 | 0 | 0 | 0 | 0 | 0 |
| 2015 | 3 | 11 | 11_03_15 #2 | -8.000 | 38.759 | 55 | Lake Lugongwe | Ruaha / Rufiji | 0 | 0 | 0 | 0 | 0 | 0 | 1 | 0 | 0 | 0 | 0 | 0 | 0 | 0 | 0 | 0 | 0 |
| 2015 | 7 | 22 | 22_07_15 #13 | -6.251 | 38.687 | 56 | Wami river | Wami river | 0 | 0 | 0 | 0 | 0 | 0 | 1 | 0 | 0 | 0 | 0 | 0 | 0 | 0 | 0 | 0 | 0 |
| 2015 | 7 | 24 | 24_07_15 #14 | -6.906 | 37.054 | 57 | Wami river | Wami river | 0 | 1 | 1 | 0 | 0 | 0 | 1 | 0 | 0 | 0 | 0 | 0 | 0 | 0 | 0 | 0 | 0 |
| 2015 | 7 | 25 | 25_07_15 #15 | -6.945 | 36.937 | 58 | Lake Nala | Wami river | 0 | 1 | 1 | 0 | 0 | 0 | 1 | 0 | 0 | 0 | 0 | 0 | 0 | 0 | 0 | 0 | 0 |
| 2015 | 7 | 26 | 26_07_15 #16 | -7.634 | 36.885 | 59 | Kidatu | Ruaha / Rufiji | 0 | 1 | 1 | 0 | 0 | 0 | 1 | 0 | 0 | 0 | 0 | 0 | 0 | 0 | 0 | 0 | 0 |
| 2015 | 7 | 31 | 31_07_15 #17 | -10.536 | 34.574 | 60 | Lituhi | Lake Malawi | 0 | 0 | 0 | 0 | 0 | 1 | 0 | 0 | 0 | 0 | 0 | 0 | 0 | 0 | 0 | 0 | 0 |
| 2015 | 8 | 1 | 01_08_15 #18 | -7.143 | 35.768 | 61 | Mtera river | Ruaha / Rufiji | 0 | 0 | 0 | 0 | 1 | 0 | 0 | 0 | 0 | 0 | 0 | 0 | 0 | 0 | 0 | 0 | 0 |
| 2015 | 8 | 11 | 11_08_15 #1 | -4.994 | 38.580 | 62 | Mayuyu dam | Pangani | 0 | 0 | 1 | 0 | 0 | 0 | 0 | 0 | 0 | 0 | 0 | 0 | 0 | 0 | 0 | 0 | 0 |
| 2015 | 8 | 12 | 12_08_15 #2 | -5.032 | 38.548 | 63 | Pangani tributory | Pangani | 0 | 1 | 1 | 0 | 0 | 0 | 0 | 1 | 0 | 0 | 0 | 0 | 0 | 0 | 0 | 0 | 0 |
| 2015 | 8 | 12 | 12_08_15 #4 | -4.886 | 38.591 | 64 | Lake Kumba | Pangani | 0 | 0 | 1 | 0 | 0 | 0 | 0 | 0 | 0 | 0 | 0 | 0 | 0 | 0 | 0 | 0 | 0 |

**Supplementary Information 1: continued**

| **Year** | **Month** | **Day** | **Site Code** | **Latitude** | **Longitude** | **Site** | **Location name** | **Catchment** | O. esculentus | O. leucostictus | O. niloticus | O. placidus | O. rukwaensis | O. shiranus | O. urolepis | O. jipe (complex) | O. amphimelas | O. korogwe | O. variabilis | O. chungruruensis | O. karomo | O. tanganicae | O. malagarasi | O. hunteri | O. "Crater lake chambo" |
| --- | --- | --- | --- | --- | --- | --- | --- | --- | --- | --- | --- | --- | --- | --- | --- | --- | --- | --- | --- | --- | --- | --- | --- | --- | --- |
| 2015 | 8 | 12 | 12_08_15 #3 | -4.806 | 38.622 | 65 | Lake Kilemele | Pangani | 1 | 1 | 1 | 0 | 0 | 0 | 0 | 1 | 0 | 0 | 0 | 0 | 0 | 0 | 0 | 0 | 0 |
| 2015 | 8 | 13 | 13_08_15 #5 | -5.068 | 38.325 | 66 | Lake Kilo | Pangani | 0 | 1 | 1 | 0 | 0 | 0 | 0 | 0 | 0 | 0 | 0 | 0 | 0 | 0 | 0 | 0 | 0 |
| 2015 | 8 | 13 | 13_08_15 #6 | -4.725 | 38.083 | 67 | Lake Manga | Pangani | 1 | 0 | 0 | 0 | 0 | 0 | 0 | 0 | 0 | 0 | 0 | 0 | 0 | 0 | 0 | 0 | 0 |
| 2015 | 8 | 13 | 13_08_15 #7 | -4.422 | 38.089 | 68 | Lake Kalimau | Pangani | 1 | 0 | 0 | 0 | 0 | 0 | 0 | 1 | 0 | 0 | 0 | 0 | 0 | 0 | 0 | 0 | 0 |
| 2015 | 8 | 14 | 14_08_15 #8 | -3.613 | 37.460 | 69 | Nyumba-ya-Mungo | Pangani | 1 | 0 | 1 | 0 | 0 | 0 | 0 | 1 | 0 | 0 | 0 | 0 | 0 | 0 | 0 | 0 | 0 |
| 2015 | 8 | 14 | 14_08_15 #11 | -3.479 | 37.589 | 70 | Kivulini fish ponds | Pangani | 0 | 0 | 1 | 0 | 0 | 0 | 0 | 0 | 0 | 0 | 0 | 0 | 0 | 0 | 0 | 0 | 0 |
| 2015 | 8 | 15 | 15_08_15 #13 | -3.890 | 37.465 | 71 | Mangulai | Pangani | 1 | 0 | 0 | 0 | 0 | 0 | 0 | 0 | 0 | 0 | 0 | 0 | 0 | 0 | 0 | 0 | 0 |
| 2015 | 8 | 15 | 15_08_15 #12 | -3.800 | 37.474 | 72 | Nyumba-ya-Mungo (National village) | Pangani | 1 | 0 | 1 | 0 | 0 | 0 | 0 | 1 | 0 | 0 | 0 | 0 | 0 | 0 | 0 | 0 | 0 |
| 2015 | 8 | 15 | 15_08_15 #14 | -3.577 | 37.737 | 73 | Lake Jipe | Pangani | 1 | 0 | 1 | 0 | 0 | 0 | 0 | 1 | 0 | 0 | 0 | 0 | 0 | 0 | 0 | 0 | 0 |
| 2015 | 8 | 16 | 16_08_15 #17 | -3.916 | 35.861 | 74 | Lake Burugi | Lake Burugi | 0 | 0 | 1 | 0 | 0 | 0 | 0 | 0 | 0 | 0 | 0 | 0 | 0 | 0 | 0 | 0 | 0 |
| 2015 | 8 | 16 | 16_08_15 #15 | -3.424 | 35.853 | 75 | Lake Manyara | Lake Manyara | 0 | 0 | 0 | 0 | 0 | 0 | 0 | 0 | 1 | 0 | 0 | 0 | 0 | 0 | 0 | 0 | 0 |
| 2015 | 8 | 16 | 16_08_15 #16 | -3.375 | 35.862 | 76 | Mto wa Mbu | Lake Manyara | 0 | 0 | 1 | 0 | 0 | 0 | 0 | 0 | 0 | 0 | 0 | 0 | 0 | 0 | 0 | 0 | 0 |
| 2015 | 8 | 18 | 18_08_15 #19 | -5.122 | 38.857 | 77 | Mlingano Dam | Mlingano dam | 0 | 0 | 0 | 0 | 0 | 0 | 0 | 0 | 0 | 1 | 0 | 0 | 0 | 0 | 0 | 0 | 0 |
| 2015 | 8 | 18 | 18_08_15 #20 | -5.042 | 38.898 | 78 | Zigi River | Zigi river | 1 | 0 | 1 | 0 | 0 | 0 | 0 | 0 | 0 | 1 | 0 | 0 | 0 | 0 | 0 | 0 | 0 |
| 2015 | 8 | 19 | 19_08_15 #23 | -6.443 | 38.845 | 79 | Ruvu river (lower) | Ruvu river | 0 | 0 | 0 | 0 | 0 | 0 | 1 | 0 | 0 | 0 | 0 | 0 | 0 | 0 | 0 | 0 | 0 |
| 2015 | 8 | 19 | 19_08_15 #22 | -5.347 | 38.645 | 80 | Pangani falls dam | Pangani | 0 | 0 | 1 | 0 | 0 | 0 | 0 | 1 | 0 | 1 | 0 | 0 | 0 | 0 | 0 | 0 | 0 |
| 2015 | 8 | 19 | 19_08_15 #21 | -5.240 | 38.700 | 81 | Makole Ziwani | Pangani | 0 | 0 | 1 | 0 | 0 | 0 | 0 | 0 | 0 | 0 | 0 | 0 | 0 | 0 | 0 | 0 | 0 |
| 2016 | 7 | 27 | 27_07_16 #2 | -4.915 | 29.611 | 82 | Katanga, Lake Tanganyika | Tanganyika/Malagarasi | 0 | 0 | 0 | 0 | 0 | 0 | 0 | 0 | 0 | 0 | 0 | 0 | 0 | 1 | 0 | 0 | 0 |
| 2016 | 7 | 27 | 27_07_16 #1 | -4.907 | 29.666 | 83 | Kagera, Ujiji (fish from Luiche river) | Tanganyika/Malagarasi | 0 | 1 | 1 | 0 | 0 | 0 | 0 | 0 | 0 | 0 | 0 | 0 | 0 | 1 | 1 | 0 | 0 |
| 2016 | 7 | 28 | 28_07_16 #3 | -5.212 | 29.842 | 84 | Malagarasi ferry | Tanganyika/Malagarasi | 0 | 1 | 1 | 0 | 0 | 0 | 0 | 0 | 0 | 0 | 0 | 0 | 0 | 1 | 1 | 0 | 0 |
| 2016 | 7 | 29 | 29_07_16 #5 | -4.860 | 29.621 | 85 | Kaseke river | Tanganyika/Malagarasi | 0 | 1 | 1 | 0 | 0 | 0 | 0 | 0 | 0 | 0 | 0 | 0 | 0 | 0 | 0 | 0 | 0 |
| 2016 | 7 | 30 | 30_07_16 #9 | -5.187 | 31.060 | 86 | Lake Sagera | Tanganyika/Malagarasi | 1 | 1 | 0 | 0 | 0 | 0 | 0 | 0 | 0 | 0 | 0 | 0 | 1 | 0 | 1 | 0 | 0 |
| 2016 | 7 | 30 | 30_07_16 #8 | -5.113 | 30.391 | 87 | Malagarasi river, Uvinza | Tanganyika/Malagarasi | 0 | 1 | 0 | 0 | 0 | 0 | 0 | 0 | 0 | 0 | 0 | 0 | 0 | 0 | 1 | 0 | 0 |
| 2016 | 7 | 30 | 30_07_16 #7 | -4.916 | 29.685 | 88 | Kigoma, fish ponds | Tanganyika/Malagarasi | 0 | 0 | 0 | 0 | 0 | 0 | 0 | 0 | 0 | 0 | 0 | 0 | 0 | 0 | 1 | 0 | 0 |
| 2016 | 7 | 31 | 31_07_16 #10 | -5.094 | 30.848 | 89 | Malagarasi river, Malagarasi rail station | Tanganyika/Malagarasi | 0 | 1 | 0 | 0 | 0 | 0 | 0 | 0 | 0 | 0 | 0 | 0 | 1 | 0 | 1 | 0 | 0 |
| 2016 | 7 | 31 | 31_07_16 #11 | -4.998 | 31.196 | 90 | Lake Nyamagoma | Tanganyika/Malagarasi | 0 | 1 | 0 | 0 | 0 | 0 | 0 | 0 | 0 | 0 | 0 | 0 | 1 | 0 | 1 | 0 | 0 |
| 2016 | 8 | 1 | 01_08_16 #12 | -5.011 | 32.892 | 91 | Kazima dam | Tanganyika/Malagarasi | 0 | 1 | 1 | 0 | 0 | 0 | 0 | 0 | 0 | 0 | 0 | 0 | 0 | 0 | 0 | 0 | 0 |
| 2016 | 8 | 2 | 02_08_16 #13 | -4.854 | 32.745 | 92 | Lake Igombe | Tanganyika/Malagarasi | 1 | 1 | 0 | 0 | 0 | 0 | 0 | 0 | 0 | 0 | 0 | 0 | 0 | 0 | 1 | 0 | 0 |
| 2016 | 8 | 2 | 02_08_16 #15 | -4.356 | 33.877 | 93 | Mwamapuli dam | Lake Eyasi | 1 | 0 | 1 | 0 | 0 | 0 | 0 | 0 | 0 | 0 | 0 | 0 | 0 | 0 | 0 | 0 | 0 |
| 2016 | 8 | 2 | 02_08_16 #14 | -4.070 | 33.789 | 94 | Igogo dam | Lake Eyasi | 0 | 0 | 1 | 0 | 0 | 0 | 0 | 0 | 0 | 0 | 0 | 0 | 0 | 0 | 0 | 0 | 0 |
| 2016 | 8 | 4 | 04_08_16 #17 | -2.643 | 32.960 | 95 | Nyashishi river | Lake Victoria | 0 | 1 | 1 | 0 | 0 | 0 | 0 | 0 | 0 | 0 | 0 | 0 | 0 | 0 | 0 | 0 | 0 |
| 2016 | 8 | 4 | 04_08_16 #16 | -2.628 | 32.898 | 96 | Lake Malimbe | Lake Victoria | 1 | 1 | 1 | 0 | 0 | 0 | 0 | 0 | 0 | 0 | 0 | 0 | 0 | 0 | 0 | 0 | 0 |
| 2016 | 8 | 4 | 04_08_16 #18 | -2.585 | 32.899 | 97 | Stream near TAFIRI, Mwanza | Lake Victoria | 0 | 1 | 0 | 0 | 0 | 0 | 0 | 0 | 0 | 0 | 0 | 0 | 0 | 0 | 0 | 0 | 0 |

**Supplementary Information 1: continued**

| **Year** | **Month** | **Day** | **Site Code** | **Latitude** | **Longitude** | **Site** | **Location name** | **Catchment** | O. esculentus | O. leucostictus | O. niloticus | O. placidus | O. rukwaensis | O. shiranus | O. urolepis | O. jipe (complex) | O. amphimelas | O. korogwe | O. variabilis | O. chungruruensis | O. karomo | O. tanganicae | O. malagarasi | O. hunteri | O. "Crater lake chambo" |
| --- | --- | --- | --- | --- | --- | --- | --- | --- | --- | --- | --- | --- | --- | --- | --- | --- | --- | --- | --- | --- | --- | --- | --- | --- | --- |
| 2016 | 8 | 5 | 05_08_16 #19 | -2.365 | 32.922 | 98 | Makobe island | Lake Victoria | 0 | 0 | 1 | 0 | 0 | 0 | 0 | 0 | 0 | 0 | 1 | 0 | 0 | 0 | 0 | 0 | 0 |
| 2016 | 8 | 6 | 06_08_16 #20 | -2.591 | 32.882 | 99 | Lake Victoria, Mwanza Gulf | Lake Victoria | 0 | 0 | 1 | 0 | 0 | 0 | 0 | 0 | 0 | 0 | 0 | 0 | 0 | 0 | 0 | 0 | 0 |
| 2016 | 10 | 3 | 03_10_16 #1 | -6.079 | 39.336 | 100 | Mgeni Haji (Uzini) Fish pond | Zanzibar | 0 | 0 | 1 | 0 | 0 | 0 | 0 | 0 | 0 | 0 | 0 | 0 | 0 | 0 | 0 | 0 | 0 |
| 2016 | 10 | 3 | 03_10_16 #2 | -6.016 | 39.245 | 101 | Mwera River (tributary of Zingwezingwe) | Zanzibar | 0 | 0 | 0 | 0 | 0 | 0 | 1 | 0 | 0 | 0 | 0 | 0 | 0 | 0 | 0 | 0 | 0 |
| 2016 | 10 | 4 | 04_10_16 #3 | -6.261 | 39.242 | 102 | Shakani Fish Farm, Zanzibar | Zanzibar | 0 | 0 | 0 | 0 | 0 | 0 | 1 | 0 | 0 | 0 | 0 | 0 | 0 | 0 | 0 | 0 | 0 |
| 2016 | 10 | 5 | 09_10_16 #4 | -5.249 | 39.776 | 103 | Kiwaleni Lake, Kibaridi, Pemba | Pemba | 0 | 0 | 0 | 0 | 0 | 0 | 1 | 0 | 0 | 0 | 0 | 0 | 0 | 0 | 0 | 0 | 0 |
| 2016 | 10 | 10 | 10_10_16 #8 | -5.950 | 39.217 | 104 | Mahonda Mwanakombo | Zanzibar | 0 | 0 | 0 | 0 | 0 | 0 | 1 | 0 | 0 | 0 | 0 | 0 | 0 | 0 | 0 | 0 | 0 |
| 2016 | 10 | 10 | 10_10_16 #5 | -5.137 | 39.807 | 105 | Kangagani freshwater pond, Pemba | Pemba | 0 | 0 | 0 | 0 | 0 | 0 | 1 | 0 | 0 | 0 | 0 | 0 | 0 | 0 | 0 | 0 | 0 |
| 2016 | 10 | 10 | 10_10_16 #6 | -5.137 | 39.807 | 106 | Kangagani saltwater pond, Pemba | Pemba | 0 | 0 | 0 | 0 | 0 | 0 | 1 | 0 | 0 | 0 | 0 | 0 | 0 | 0 | 0 | 0 | 0 |
| 2016 | 10 | 10 | 10_10_16 #7 | -5.137 | 39.807 | 107 | Saltwater pond at Pujini, Pemba | Pemba | 0 | 0 | 0 | 0 | 0 | 0 | 1 | 0 | 0 | 0 | 0 | 0 | 0 | 0 | 0 | 0 | 0 |
| 2016 | 10 | 21 | 21_10_16 #1 | -10.044 | 39.505 | 108 | Lake Mitupa | Rutamba lakes | 0 | 0 | 1 | 0 | 0 | 0 | 0 | 0 | 0 | 1 | 0 | 0 | 0 | 0 | 0 | 0 | 0 |
| 2016 | 10 | 24 | 24_10_16 #3 | -10.044 | 39.453 | 109 | Lake Nambawala | Rutamba lakes | 0 | 0 | 1 | 0 | 0 | 0 | 0 | 0 | 0 | 1 | 0 | 0 | 0 | 0 | 0 | 0 | 0 |
| 2016 | 10 | 25 | 25_10_16 #4 | -9.567 | 39.603 | 110 | Mkoe lake | Mbwenkuru | 0 | 0 | 1 | 0 | 0 | 0 | 1 | 0 | 0 | 0 | 0 | 0 | 0 | 0 | 0 | 0 | 0 |
| 2016 | 11 | 1 | 01_11_16 #1 | -6.904 | 39.119 | 111 | Dar-es-Salaam | Dar-es-Salaam | 0 | 0 | 1 | 0 | 0 | 0 | 0 | 0 | 0 | 0 | 0 | 0 | 0 | 0 | 0 | 0 | 0 |
| 2017 | 1 | 11 | 11_01_17 #S | -6.124 | 35.297 | 112 | Lake Sulungali | Lake Sulungali | 0 | 0 | 1 | 0 | 0 | 0 | 0 | 0 | 1 | 0 | 0 | 0 | 0 | 0 | 0 | 0 | 0 |
| 2017 | 7 | 23 | 23_07_17 #2 | -9.013 | 33.011 | 113 | Mlowo river | Lake Rukwa | 0 | 0 | 0 | 0 | 1 | 0 | 0 | 0 | 0 | 0 | 0 | 0 | 0 | 0 | 0 | 0 | 0 |
| 2017 | 7 | 23 | 23_07_17 #5 | -9.315 | 32.769 | 114 | Tunduma fish ponds | Lake Rukwa | 0 | 0 | 1 | 0 | 0 | 0 | 0 | 0 | 0 | 0 | 0 | 0 | 0 | 0 | 0 | 0 | 0 |
| 2017 | 7 | 24 | 24_07_17 #7 | -8.354 | 32.285 | 115 | Kilyamatundu market (from Lake Rukwa) | Lake Rukwa | 1 | 0 | 0 | 0 | 1 | 0 | 0 | 0 | 0 | 0 | 0 | 0 | 0 | 0 | 0 | 0 | 0 |
| 2017 | 7 | 24 | 24_07_17 #8 | -8.241 | 32.439 | 116 | Changani landing beach | Lake Rukwa | 1 | 0 | 0 | 0 | 1 | 0 | 0 | 0 | 0 | 0 | 0 | 0 | 0 | 0 | 0 | 0 | 0 |
| 2017 | 7 | 25 | 26_07_17 #14 | -8.403 | 31.975 | 117 | Lake Kwela | Lake Rukwa | 0 | 0 | 0 | 0 | 1 | 0 | 0 | 0 | 0 | 0 | 0 | 0 | 0 | 0 | 0 | 0 | 0 |
| 2017 | 7 | 27 | 27_07_17 #15 | -7.942 | 31.596 | 118 | Lwiche river at Sumbawanga | Lake Rukwa | 0 | 0 | 0 | 0 | 1 | 0 | 0 | 0 | 0 | 0 | 0 | 0 | 0 | 0 | 0 | 0 | 0 |
| 2017 | 7 | 27 | 27_07_17 #17 | -8.534 | 31.641 | 119 | Lake Sundu | Lake Tanganyika | 0 | 1 | 0 | 0 | 0 | 0 | 0 | 0 | 0 | 0 | 0 | 0 | 0 | 0 | 0 | 0 | 0 |
| 2017 | 7 | 27 | 27_07_17 #19 | -8.071 | 31.575 | 120 | Sumbawanga fish ponds | Lake Rukwa | 0 | 0 | 1 | 0 | 0 | 0 | 0 | 0 | 0 | 0 | 0 | 0 | 0 | 0 | 0 | 0 | 0 |
| 2017 | 7 | 27 | 28_07_17 #21 | -6.323 | 31.049 | 121 | Milala Dam | Lake Rukwa | 0 | 0 | 0 | 0 | 1 | 0 | 0 | 0 | 0 | 0 | 0 | 0 | 0 | 0 | 0 | 0 | 0 |
| 2017 | 7 | 29 | 29_07_17 #25 | -6.365 | 31.247 | 122 | Msaginya river | Lake Rukwa | 0 | 1 | 0 | 0 | 1 | 0 | 0 | 0 | 0 | 0 | 0 | 0 | 0 | 0 | 0 | 0 | 0 |
| 2017 | 7 | 30 | 30_07_17 #26 | -6.352 | 31.074 | 123 | Kaushauliili fish ponds | Lake Rukwa | 0 | 1 | 0 | 0 | 0 | 0 | 0 | 0 | 0 | 0 | 0 | 0 | 0 | 0 | 0 | 0 | 0 |
